# Advanced mandibular reconstruction with fibular free flap and alloplastic TMJ prosthesis with digital planning

**DOI:** 10.1186/s40463-023-00639-4

**Published:** 2023-07-03

**Authors:** Justin M. Pyne, Clayton M. Davis, Ryan Kelm, Claudine Bussolaro, Walter Dobrovolsky, Hadi Seikaly

**Affiliations:** 1 Division of Otolaryngology - Head and Neck Surgery, University of Alberta Hospital, University of Alberta, 8440 112 Street NW, Edmonton, AB T6G 2B7 Canada; 2 Division of Oral and Maxillofacial Surgery, Faculty of Medicine and Dentistry, University of Alberta Hospital, University of Alberta, 8440 112 Street NW, Edmonton, AB T6G 2B7 Canada

**Keywords:** Jaw reconstruction, Dental rehabilitation, Oral and maxillofacial surgery, Oral neoplasm, Temporomandibular joint prosthesis, Temporomandibular joint dysfunction, Surgical design, Simulation

## Abstract

**Introduction:**

Resection of the mandible and temporomandibular joint (TMJ) without formal reconstruction is a devastating condition that negatively affects all aspects of the patient’s life. We have approached the reconstruction of mandibular defects that include the condyle with simultaneous reconstruction with a vascularized free fibular flap (FFF) using Surgical Design and Simulation (SDS) and alloplastic TMJ prosthesis. The objective of this study is to report the functional and quality of life (QOL) outcomes in a cohort of patients that had undergone our reconstructive protocol.

**Methods:**

This was a prospective case series of adult patients that underwent mandibular reconstruction with FFF and alloplastic TMJ prosthesis at the our center. Pre-operative and post-operative maximum inter-incisal opening (MIO) measurements were collected, and patients completed a QOL questionnaire (EORTC QLQ—H&N35) during those perioperative visits.

**Results:**

Six patients were included in the study. The median patient age was 53 years. Heat map analysis of the QOL questionnaire revealed that patients reported a positive clinically significant change in the domains of pain, teeth, mouth opening, dry mouth, sticky saliva, and senses (relative change of 2.0, 3.3, 3.3, 2.0, 2.0, and 1.0 respectively). There were no negative clinically significant changes. There was a median perioperative MIO increase of 15.0 mm, and this was statistically significant (*p*  =  0.027).

**Conclusions:**

This study highlights the complexities involved in mandibular reconstruction with involvement of the TMJ. Based on our findings, patients can obtain an acceptable QOL and good function following simultaneous reconstruction with FFF employing SDS and an alloplastic TMJ prosthesis.

**Graphical Abstract:**

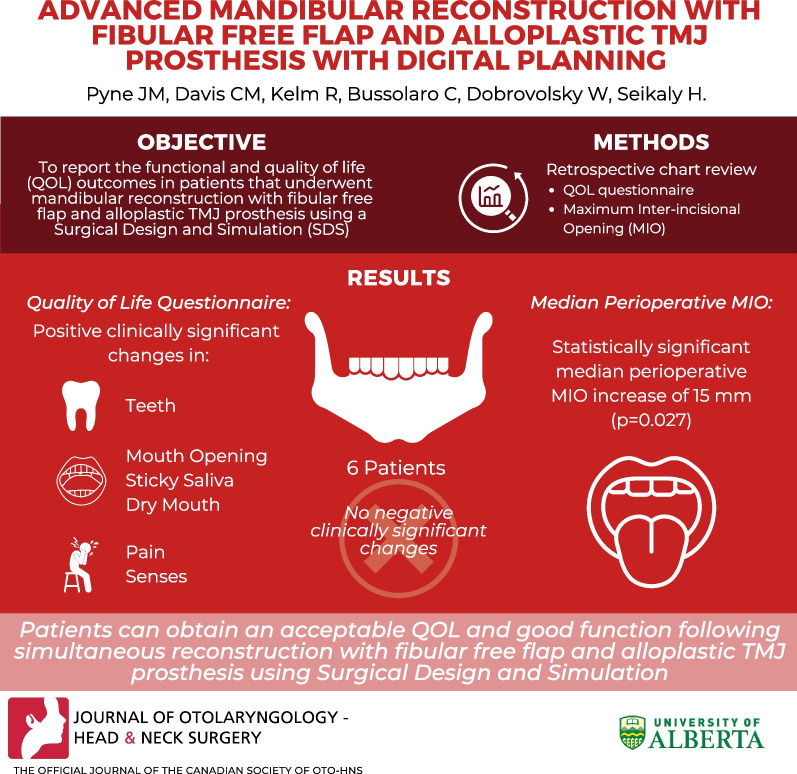

## Introduction

Resection of the mandible and temporomandibular joint (TMJ) without formal reconstruction is a devastating condition that negatively affects all aspects of the patient’s life. Many reconstructive techniques have been developed to address these complex defects with an overarching purpose of restoring function, cosmesis and quality of life in this patient population [1]. The main principles of these reconstructive protocols include the maintenance of the jaw continuity, maintenance of joint alignment and integrity, restoration of occlusion, and recreation of the facial contour.

**Fig. 1:**
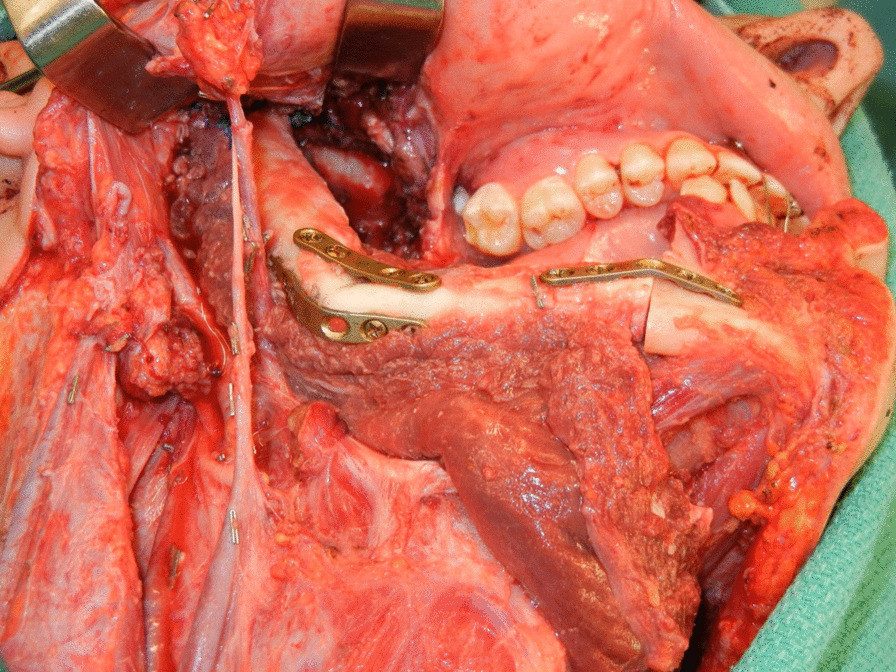
Fibula free flap reconstruction with contouring of the distal end to allow for the fibula to fit passively into the glenoid fossa with anchoring to the remnant joint capsule or disc

**Fig. 2:**
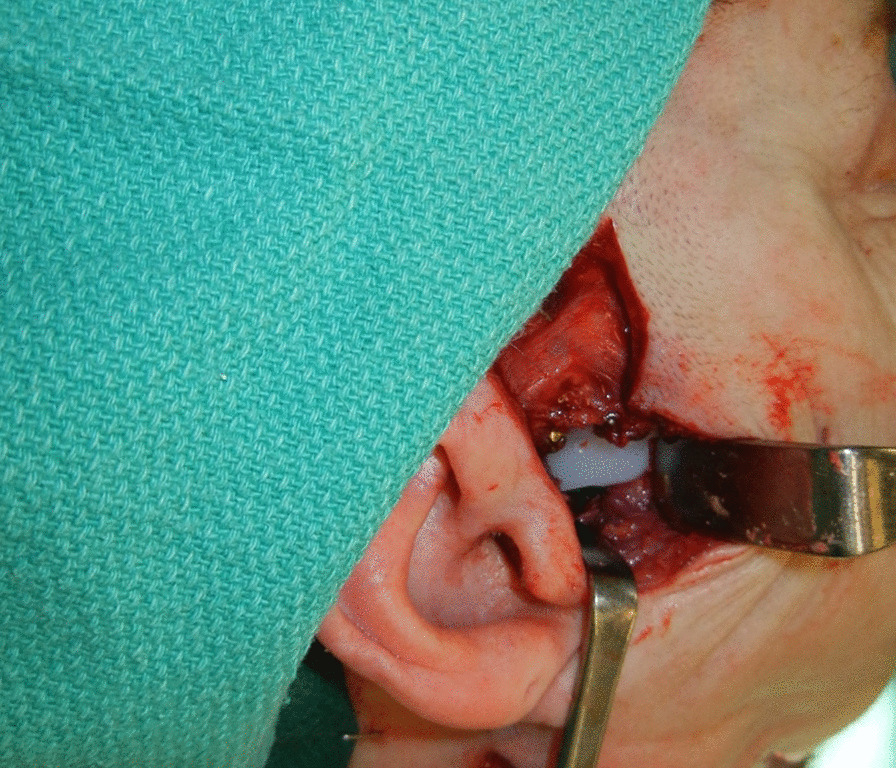
The fibula and fossa reconstructed was secured to the residual mandible with a Synthes 2 mm plate. Once this was completed, an additional 3 or 4 screws were placed to hold the fossa prosthesis in place

**Fig. 3:**
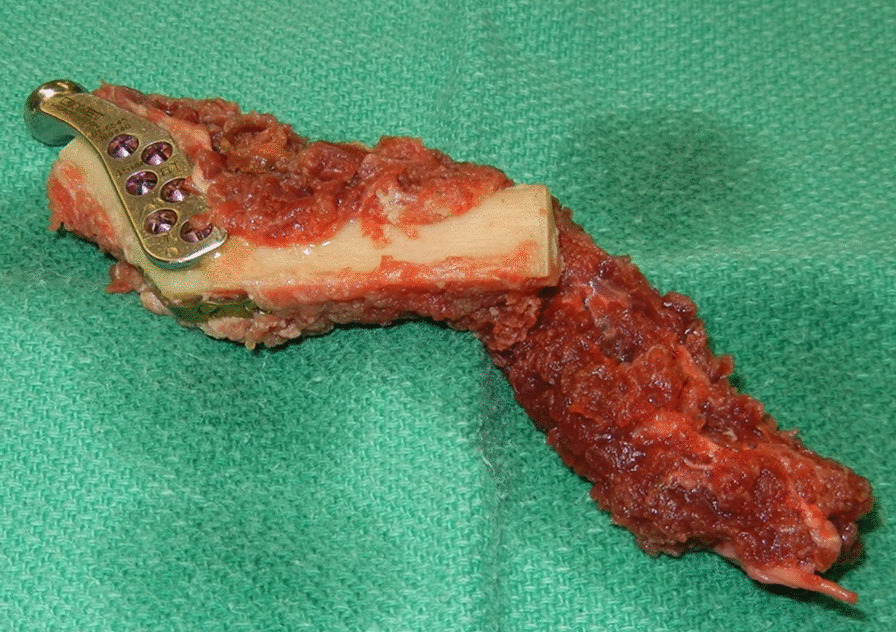
Stock alloplastic TMJ prosthetic joint replacement plated to FFF. L-shaped reconstruction of the mandible with the fibula was fashioned. The condylar prosthesis was adapted to the fibula and secured in place, and a narrow 45 or 50 mm stock Biomet prosthesis was normally used

**Fig. 4:**
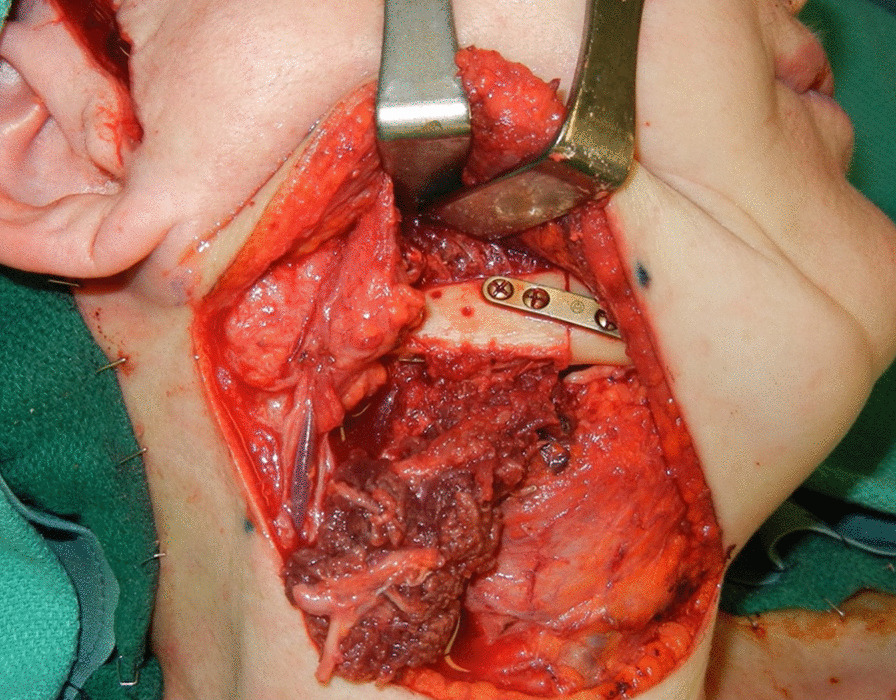
Reconstructive construct of the fibula and the alloplastic joint is anchored in place with the masseter muscle and parotid fascia reattachment to the FFF. Plating of the FFF to native mandible is also seen

The standard of care of modern mandibular reconstruction are free tissue transfer techniques. These procedures have a proven record of restoring function and significantly improving the patients’ cosmetic outcomes [1, 2]. The reconstruction of the temporomandibular joint (TMJ), however, continues to be one of the most difficult and complicated reconstructive challenges [3]. The options range from no TMJ reconstruction, plating of the condyle on the flap bone, fabrication and attachment of the free flap reconstruction to the remnant joint capsule or disc (Fig. 1), to prosthetic joint replacement (Figs. 2, 3, 4) [4].

Free flap reconstructive protocols for the mandible have been refined over the years, and presently the osteocutaneous fibula free flap (FFF) is considered one of the best options for mandibular reconstruction [4–7]. The FFF yields the least amount of resorption and is more stable in the long term in comparison to the iliac crest free flap (ICFF) and the scapula free flap (SFF) [6]. The contouring of the fibula allows for a three-dimensional reconstruction of the mandible, providing up to 26 cm of bone serving as a scaffold for the placement of dental implants if necessary [4]. When the mandibular resection includes the condyle, reconstructive techniques of plating the condyle on the flap bone, fabricating the fibular bone and attaching it to the remnant joint capsule and disc have the major disadvantage of degeneration and ankylosis secondary to heterotopic ossification [8–14]. This unfortunate complication can lead to loss of function and a severely hypomobile mandible [15–18].

We have approached the reconstruction of mandibular defects that include the condyle by employing simultaneous reconstruction with a vascularized FFF using Surgical Design and Simulation (SDS) and alloplastic TMJ prosthesis. The goal of TMJ total joint replacement surgery was to restore the patients to full function and maintain their quality of life (QOL). There are currently two FDA-approved alloplastic TMJ total joint replacement systems available in the USA: the stock Total Mandibular Joint Replacement System by Zimmer Biomet® (Warsaw, IN) and the custom fit TMJ Concepts® TMJ Reconstruction Prosthesis System (Ventura, CA) [19]. TMJ Concepts® was recently purchased by Stryker® and the two have essentially become one company.

According to the American Association of Oral and Maxillofacial Surgeons (AAOMS), indications for TMJ replacement surgery include failure of satisfactory outcomes following repeated operations on the TMJ, persistent functionally limiting symptoms despite attempted conservative therapies, TMJ destruction due to connective tissue, autoimmune, inflammatory, infective, or reactive diseases, TMJ ankylosis, failed reconstruction with autogenous grafts, and neoplasia [20].

There are several case reports describing outcomes of simultaneous FFF with alloplastic TMJ prosthesis [21, 22], but the literature is lacking a long-term series of fibular free flap and alloplastic joint replacement in the reconstruction of large mandibular defects that assess the patients functional and quality of life outcomes. The objective of this study is to report the functional and QOL outcomes on a cohort of patients that had undergone FFF with SDS and an alloplastic TMJ prosthesis employing the stock Biomet® joint.

## Materials and methods

### Study design

This was a prospective case series of adult patients who underwent resection of the mandible with condylectomy and mandibular reconstruction with FFF and stock alloplastic TMJ prosthesis between August 2004 and December 2022. Ethics approval was obtained under number Pro00087350. Surgical teams from the Division of Oral and Maxillofacial Surgery and the Division of Otolaryngology—Head and Neck Surgery at the University of Alberta were involved in all aspects of patient management. The manuscript was prepared according to the appropriate EQUATOR guidelines. We have read the Helsinki Declaration and have followed the guidelines in this investigation.

All adult patients who underwent mandible reconstruction with FFF and alloplastic TMJ prosthesis at the University of Alberta were assessed. We included patients that:

1. Underwent simultaneous reconstruction with a FFF and an alloplastic stock Biomet® TMJ prosthesis.
2. Had been followed in the interdisciplinary Head and Neck Surgery Functional Assessment Laboratory (HNSFAL) at the Institute for Reconstructive Sciences (iRSM).

### Surgical technique

For all jaw reconstructions, preoperative planning was undertaken with an SDS methodology. This technique involves utilization of a pre-operative high resolution helical computed tomography (CT) scan of the facial bones with subsequent CT scan of the fibular flap donor site based on a previously developed protocol at our center [23, 24]. The CT images were converted into three-dimensional renderings which were then analyzed virtually and used to develop the surgical plan. Digitally designed and additive manufactured surgical drilling and cutting guides were fashioned from the virtual models and translated to a physical model which could be sterilized and used intraoperatively.

### TMJ preparation

Fossa was recontoured with rotary instruments such as a mastoid bur or custom rasp to allow for passive seating of the fossa prostheses. Once this aspect was completed, a fossa prosthesis was adapted and secured in place with a two fossa screws. An additional 3 or 4 screws were then placed to hold the fossa prosthesis in place (Fig. 2).

### TMJ reconstruction

An L-shaped reconstruction of the mandible with the fibula was fashioned. The condylar prosthesis was adapted to the fibula and secured in place, and a narrow 45 or 50 mm stock Biomet prosthesis was normally used (Fig. 3). In one instance, the prosthesis itself was used to secure to horizontal component of the FFF to its vertical component. Following completion of TMJ and fibula placement, the construct was held in place with the reattachment of the masseter muscle and parotid gland fascia to the FFF. When these structures were deficient, tensor fascia was harvested and attached to the zygoma superiorly and onto the bone of the fibula inferiorly. These maneuvers ensured that the condyle was well anchored in the fossa. (Fig. 4).

### Objective measures

Each patient underwent pre-operative and post-operative assessments. Maximum inter-incisal opening (MIO) measurements were collected at each appointment.

### Subjective measures

The EORTC QLQ—H&N35 questionnaire [25–28] was completed by patients during their assessments. Quality of life (QOL) domains that patients perceive as the most affected following surgery were identified. Measured on a scale from 0 to 100, a lower score corresponds with a subjective improvement in the patient's QOL while a higher score indicates an unfavourable outcome.

### Statical analysis

Statistical analysis was performed using IBM SPSS 25 (Armonk, NY). We calculated descriptive statistics and compared pre-operative and post-operative maximal inter-incisal opening results using non-parametric testing (Wilcoxon signed-rank test.) A *p*-value of < 0.05 was considered statistically significant. Several methods exist that are used to report the MCID [29–31]. A systematic review by Michaelsen et al. reviewing quality of life in head and neck cancer patients concluded that the most studies accepted a 10% change of the maximal score as corresponding to the MCID [31], which is also supported elsewhere in the literature [32–35]. Thus, this same principle was adopted for the purposes of this study. Further, we have incorporated the same heat map analysis utilized in their systematic review where “clinically important differences" are presented in intervals of 10% of the maximal instrument score. For each domain, the relative QOL difference was calculated as the absolute difference between the QOL score at follow-up and the reference score, divided by the MCID. We refer to differences in QOL constituting 10–19.9% of the maximal instrument score as minimal (ratio 1.00–1.99), 20–29.9% differences as moderate (ratio 2.00–2.99), and ≥ 30% differences as large clinically important differences (ratio ≥ 3.00). For ease of interpretation, ratios that designate improvements in function have been assigned positive values, whereas negative values designate deteriorations [32].

## Results

Six patients were included in the study. (3 females (50.0%), 3 males (50.0%)). The median patient age was 53 years. Five patients completed the EORTC QLQ—H&N35 questionnaire preoperatively and were included in the final quality of life calculations. The median follow-up time was 14.38 years (range 4.03–17.43 years). Review of complications identified one major and one minor complication, with no implant losses overall. Complete patient demographics are listed in Table 1.

**Table 1:** Patient demographics, pathology, and complications

Number of subjects	6
Female	3 50.0%)
Male	3 (50.0%)
Age (years; range)	53; 33–85
*Pathology*	
Benign	6 (100.0%)
TMJ erosion	1 (16.7%)
Ameloblastoma	2 (33.3%)
Osteoblastoma	1 (16.7%)
Osteomyelitis	1 (16.7%)
Osteoradionecrosis	1 (16.7%)
*Complications*	
Major (infection, successfully treated with IV antibiotics)	1 (16.7%)
Minor (displacement of the prosthetic head from the glenoid fossa, successfully reduced)	1 (16.7%)

Quality of life outcomes were assessed preoperatively and postoperatively using the EORTC QLQ-H&N-35 questionnaire. A heat map analysis revealed that patients reported a positive clinically significant change in the domains pain, teeth, mouth opening, dry mouth, sticky saliva, and senses (relative change of 2.0, 3.3, 3.3, 2.0, 2.0, and 1.0 respectively). There were no negative clinically significant changes. No patients required a feeding tube postoperatively. Results are highlighted in Table 2. MIO was recorded perioperatively for all patients. The preoperative median MIO was 15.5 mm while the postoperative median MIO was 30.5 mm, yielding a median increase of 15.0 mm, and this was statistically significant (*p* = 0.027) (Table 3).

**Table 2:** Perioperative median EORTC QLQ-H&N-35 scores, calculated MCID, and heat map analysis

Value		Preoperative		Postoperative		Temporal QOL Change/MCID^∤^
		Median score		Median score		Relative change^⨎^
Pain		36.7		16.7		2.0^→^
Swallow		31.7		35.0		−0.3^⥈^
Teeth		60.0		26.7		3.3^⟹^
Mouth opening		80.0		46.7		3.3^⟹^
Dry mouth		80.0		60.0		2.0^→^
Sticky saliva		40.0		20.0		2.0^→^
Senses		26.7		16.7		1.0^⇢^
Coughing		26.7		33.3		−0.7^⥈^
Felt Ill		26.7		26.7		0.0^⥈^
Speech		35.6		40.0		−0.4^⥈^
Social eating		53.3		50.0		0.3^⥈^
Contact		33.3		28.0		0.5^⥈^
Sexuality		33.3		37.5		−0.4^⥈^
Pain killers		20.0		26.7		−0.7^⥈^
Nutritional supplementation		20.0		20.0		0.0^⥈^
Feeding tube		6.7		0.0		0.7^⥈^
Weight loss		20.0		26.7		−0.7^⥈^
Weight gain		0.0		0.0		0.0^⥈^

Heat map spectrum						
⟸	←	⇠	⥈	⇢	→	⟹
≤ −3	−2	−1	0	1	2	≥ 3
Negative clinically significant change (deterioation in function)			No clinically significant change	Positive clinically significant change (improvement in function)		

^∤^Minimal clinically important difference equals 10% of maximal instrument score

⨎ Heat map: for each domain, the relative QOL difference was calculated as the absolute difference between quality-of-life score preoperatively and quality of life score postoperatively, divided by the MCID. Our study refers to differences in quality of life constituting 10 to 19.9% of the maximal instrument score as minimal (ratio 1.00–1.99), 20 to 29.9% differences as moderate ratio (2.00–2.99), and ≥ 30% differences as large clinically important differences (ratio ≥ 3.00. To ease interpretation, ratios that designate improvements in function have been assigned positive values whereas negative values designate deterioration. Adapted from Michaelsen et al. Clinically important. [31]

**Table 3:** Perioperative maximal inter-incisal opening values

	Preoperative	Postoperative	Median difference	*p*
Maximal inter-incisal opening (mm)	15.5	30.5	15	0.027

## Discussion

This study is the largest reported single-center cohort of patients undergoing simultaneous mandibular reconstruction with FFF employing SDS and an alloplastic TMJ prosthesis. The present findings demonstrate that patients with mandibular defects that include the condyle can be effectively reconstructed. Our patient cohort achieved a statistically significant improvement in their jaw opening. The patients also had clinically significant improvement in their quality of life as demonstrated by the EORTC QLQ-H&N-35 questionnaire. The heat map analysis revealed that patients had positive clinically significant change in the domains of pain, teeth, mouth opening, dry mouth, sticky saliva, and senses with no negative clinically significant changes. Improvements in the teeth and mouth opening domains may be attributable directly to the improved jaw opening postoperatively, allowing the patient to utilize their teeth more effectively. Decreased pain post-operatively is likely due to the removal of benign, function limiting pathologies whereas improved dry mouth symptoms are likely related to the increased oral intake.

There are a variety of possible complications related to TMJ joint reconstruction with alloplastic materials in the setting of concurrent mandible reconstruction. Reported complications in joint reconstruction procedures include chronic pain, joint instability, and trismus as well as decreased interincisal opening secondary to radiotherapy and the extent of resection [15, 36]. Further, alloplastic TMJ prosthesis cases are associated with increased sensitivity or allergy to metals, implant failure, malocclusion and infections [37, 38]. When free flap mandibular reconstructive surgery is performed in addition joint reconstruction, this can expose the patient to further risks such as significant bleeding, osteomyelitis, bony non-union, tissue resorption, heterotropic bone formation and airway obstruction, with these patients typically undergoing a prophylactic tracheostomy to prevent the latter [39, 40]. Fortunately, there were only two reported complications within the cohort: one major (joint infection requiring IV antibiotic therapy, which subsequently resolved) and one minor (displacement of the prosthetic head from the glenoid fossa, successfully reduced).

Several methods of TMJ reconstruction following mandible and condyle resection have been described, each with varying results. Contouring the distal end of the FFF to allow for the fibula to fit passively into the glenoid fossa (Fig. 1) is one solution well described in the literature. Wax et al. assessed 17 patients who underwent FFF reconstruction of the TMJ and concluded that microvascular fibular flaps are an adequate method to reconstruct the TMJ, with most patients returning to normal function [15]. However, their study was retrospective with a shorter follow up period than our cohort. Further, their method involves suturing the fibular into the glenoid fossa, which may result in the rare, but devastating complications of ankylosis and lack of translation of the neocondyle due to decreased joint mobility and scar formation in the longer term [41]. Garcia-Gonzales et al. who assessed six patients who underwent mandibular resection involving the condyle with FFF reconstruction. The authors concluded that positioning the fibula flap directly into the glenoid fossa constitutes a reliable method for condylar reconstruction, but similarly warned of complications such as ankylosis 4. Additional outcomes measures that could be collected include swallowing with return to the level of oral intake similar to before the procedure, facial symmetry and intelligible speech, although there is variability in what is reported between centers [39, 40].

Reconstruction of mandibular defects is a complicated task for the surgical team. Furthermore, when there is involvement of the mandibular condyle, the difficulty of the reconstruction increases dramatically. The use of fibular free flaps in conjunction with alloplastic temporomandibular joint prostheses is relatively novel and requires a great deal of surgical planning and technical expertise to garner continued success. Thus, an experienced team joining Oral and Maxillofacial surgeons with reconstructive surgeons offers the patient the best chance for a functional reconstruction. Our findings are in keeping with previous work of TMJ total joint replacement surgery where alloplastic TMJ replacement systems have demonstrated safety and efficacy in the treatment of end-stage TMJ disease. These studies show significant improvement in quality of life, mandibular range of motion, function and speech while decreasing pain and dietary restrictions [42–44].

The use of FFF employing SDS and an alloplastic TMJ prosthesis results in similar functional outcomes and may avoid the common pitfalls associated with the other reconstructive methods, such as ankylosis, mispositioning, or instability. Our patient cohort maintained good long-term mandibular range of motion over a median follow-up of 36 months. While we recognize that it can be difficult to draw concrete conclusions from a small cohort, this study may help to guide the TMJ surgeon when counseling patients on what can be expected with this type of reconstruction and assist them with making an informed treatment decision.

## Conclusion

In conclusion, this study highlights the complexities involved in mandibular reconstruction with involvement of the TMJ. Based on our findings, patients can obtain an acceptable QOL and good function following simultaneous reconstruction with FFF employing SDS and an alloplastic TMJ prosthesis. Future study may include the implementation of custom digitally planned TMJ prosthetics in conjunction with an SDS planned FFF.

## Data Availability

The datasets used and/or analysed during the current study are available from the corresponding author on reasonable request.

## Abbreviations

FFF: Fibular Free Flap
TMJ: Temporal Mandibular Joint
SDS: Surgical Design and Simulation
QOL: Quality of Life
CT: Computed Tomography
MIO: Maximum Inter-incisal opening
AAOMS: American Association of Oral and Maxillofacial Surgeons
HNSFAL: Head and Neck Surgery Functional Assessment Laboratory
iRSM: Institute for Reconstructive Sciences in Medicine

## References

[1] Pyne JM, Dziegielewski PT, Constantinescu G, Dzioba A, O'Connell DA, Côté DW, Ansari K, Harris J, Conrad D, Makki FM, Hearn M, Biron VL, Seikaly H (2020). The functional & quality of life outcomes of total glossectomy with laryngeal preservation. Laryngosc Investig Otolaryngol.

[2] Dziegielewski PT, Ho ML, Rieger J, Singh P, Langille M, Harris JR, Seikaly H (2013). Total glossectomy with laryngeal preservation and free flap reconstruction: objective functional outcomes and systematic review of the literature. Laryngoscope.

[3] Granquist EJ, Quinn PD. Atlas of temporomandibular joint surgery. In: Granquist EJ, Quinn PD, editors. Second edition. ed.

[4] Erovic BM. Manual of head and neck reconstruction using regional and free flaps. In: Lercher P, editor.2014.

[5] Patel SY, Kim DD, Ghali GE (2019). Maxillofacial reconstruction using vascularized fibula free flaps and endosseous implants. Oral Maxillofac Surg Clin North Am.

[6] Wilkman T, Apajalahti S, Wilkman E, Törnwall J, Lassus P (2017). A comparison of bone resorption over time: an analysis of the free scapular, iliac crest, and fibular microvascular flaps in mandibular reconstruction. J Oral Maxillofac Surg.

[7] Ariga P, Narayanan V, Jain AR, Philip JM, Nathan S (2017). Clinical and functional outcomes of implant prostheses in fibula free flaps. World J Dent.

[8] Mays AC, Gillenwater AM, Garvey PB (2018). Rare presentation of heterotopic ossification along a fibula free flap pedicle in a high-volume microvascular reconstruction practice. Head Neck.

[9] Smith RB, Funk GF (2003). Severe trismus secondary to periosteal osteogenesis after fibula free flap maxillary reconstruction. Head Neck.

[10] Gangidi SR, Courtney D (2013). “You reap what you sow”—a case of heterotopic ossification within a fasciocutaneous radial forearm free flap reconstruction. Int J Oral Maxillofac Surg.

[11] Myon L, Ferri J, Genty M, Raoul G (2012). Consequences of bony free flap’s pedicle calcification after jaw reconstruction. J Craniofac Surg.

[12] Wood CB, Rohde SL, Sinard RJ, Mannion K, Bigcas JLM (2020). Incidence of pedicle ossification in osseous free flap reconstruction in the head and neck. Oral Oncol.

[13] Tarsitano A, Sgarzani R, Betti E, Oranges CM, Contedini F, Cipriani R, Marchetti C (2013). Vascular pedicle ossification of free fibular flap: Is it a rare phenomenon? Is it possible to avoid this risk?. Acta Otorhinolaryngol Italica.

[14] Drew SJ, Cho JS (2022). Fibula free flap reconstruction of the maxilla leading to extracapsular ankylosis of the mandible. J Oral Maxillofac Surg.

[15] Wax MK, Winslow CP, Hansen J, MacKenzie D, Cohen J, Andersen P, Albert T (2000). A retrospective analysis of temporomandibular joint reconstruction with free fibula microvascular flap. Laryngoscope.

[16] Guyot L, Richard O, Layoun W, Cheynet F, Bellot-Samson V, Chossegros C, Blanc J-L, Gola R (2004). Long-term radiological findings following reconstruction of the condyle with fibular free flaps. J Cranio-Maxillofac Surg.

[17] Engroff SL (2005). Fibula flap reconstruction of the condyle in disarticulation resections of the mandible: a case report and review of the technique. Oral Surg Oral Med Oral Pathol Oral Radiol Endodontol.

[18] González-García R, Naval-Gías L, Rodríguez-Campo FJ, Martínez-Chacón JL, Gil-Díez Usandizaga JL (2008). Vascularized fibular flap for reconstruction of the condyle after mandibular ablation. J Oral Maxillofac Surg.

[19] Yo A, McKay J, Lebovic G, Psutka DJ (2019). Temporomandibular joint total replacement using the Zimmer Biomet Microfixation patient-matched prosthesis results in reduced pain and improved function. Oral Surg Oral Med Oral Pathol Oral Radiol.

[20] Koslin MG, Indresano AT, Mercuri LG (2012). Temporomandibular Joint Surgery. J Oral Maxillofac Surg.

[21] Landes C, Korzinskas T, Dehner J-F, Santo G, Ghanaati S, Sader R (2014). One-stage microvascular mandible reconstruction and alloplastic TMJ prosthesis. J Cranio-Maxillofac Surg.

[22] Infante-Cossio P, Torres-Lagares D, Martinez-de-Fuentes R, Garcia-Perla-Garcia A, Gutierrez-Perez JL (2006). Dental restoration with endosseous implants after mandibular reconstruction using a fibula free flap and TMJ prosthesis: a patient report. Int J Oral Maxillofac Implants.

[23] Paley MD, Lloyd CJ, Penfold CN (2005). Total mandibular reconstruction for massive osteolysis of the mandible (Gorham–Stout syndrome). Br J Oral Maxillofac Surg.

[24] Jaeschke R, Singer J, Guyatt GH (1989). Measurement of health status. Ascertaining the minimal clinically important difference. Control Clin Trials.

[25] Seikaly H, Idris S, Chuka R, Jeffery C, Dzioba A, Makki F, Logan H, O’Connell DA, Harris J, Ansari K, Biron V, Cote D, MDent MO, Nayar S, Wolfaardt J (2019). The Alberta reconstructive technique: an occlusion-driven and digitally based jaw reconstruction. Laryngoscope.

[26] Singer S, Arraras J, Chie WC, Fisher S, Galalae R, Hammerlid E, Nicolatou-Galitis O, Schmalz C, Verdonck-de Leeuw IM, Gamper E, Keszte J, Hofmeister D (2013). Performance of the EORTC questionnaire for the assessment of quality of life in head and neck cancer patients EORTC QLQ-H&N35. A methodological review. Qual Life Res.

[27] Bjordal K, De Graeff A, Fayers PM, Hammerlid E, van Pottelsberghe C, Curran D, Ahlner-Elmqvist M, Maher EJ, Meyza JW, Brédart A, Söderholm AL, Arraras JJ, Feine JS, Abendstein H, Morton RP, Pignon T, Huguenin P, Bottomly A, Kaasa S (2000). A 12 country field study of the EORTC QLQ-C30 (version 3.0) and the head and neck cancer specific module (EORTC QLQ-H&N35) in head and neck patients. Eur J Cancer.

[28] Pyne JM, Kelly BC, Logan H, Osswald M, Nayar S, Biron VL, Ansari K, Harris JR, O'Connell DA, Seikaly H (2022). The modified Alberta reconstructive technique: a prospective cohort study. Oral Oncol.

[29] Lydick E, Epstein RS (1993). Interpretation of quality of life changes. Qual Life Res.

[30] Norman GR, Sloan JA, Wyrwich KW (2003). Interpretation of changes in health-related quality of life: the remarkable universality of half a standard deviation. Med Care.

[31] Høxbroe Michaelsen S, Grønhøj C, Høxbroe Michaelsen J, Friborg J, von Buchwald C (2017). Quality of life in survivors of oropharyngeal cancer: a systematic review and meta-analysis of 1366 patients. Eur J Cancer.

[32] Ringash J, O'Sullivan B, Bezjak A, Redelmeier DA (2007). Interpreting clinically significant changes in patient-reported outcomes. Cancer.

[33] Osoba D, Rodrigues G, Myles J, Zee B, Pater J (1998). Interpreting the significance of changes in health-related quality-of-life scores. J Clin Oncol.

[34] Barrett B, Brown D, Mundt M, Brown R (2005). Sufficiently important difference: expanding the framework of clinical significance. Med Decis Making.

[35] Hutcheson KA, Barrow MP, Lisec A, Barringer DA, Gries K, Lewin JS (2016). What is a clinically relevant difference in MDADI scores between groups of head and neck cancer patients?. Laryngoscope.

[36] Hidalgo DA (1994). Condyle transplantation in free flap mandible reconstruction. Plast Reconstr Surg.

[37] Tiongco RP, Hui A, Stern-Buchbinder Z, Stalder MW, St. Hilaire H, (2021). Reconstruction of bilateral mandibular condyles using a single vascularized fibula. Plast Reconstr Surg Glob Open.

[38] Tarsitano A, Battaglia S, Ramieri V, Cascone P, Ciocca L, Scotti R (2017). Short-term outcomes of mandibular reconstruction in oncological patients using a CAD/CAM prosthesis including a condyle supporting a fibular free flap. J Cranio-Maxillofacial Surg.

[39] Lee ZH, Avraham T, Monaco C, Patel AA, Hirsch DL, Levine JP (2018). Optimizing functional outcomes in mandibular condyle reconstruction with the free fibula flap using computer-aided design and manufacturing technology. J Oral Maxillofac Surg.

[40] Marx RE, Cillo JE, Broumand V, Ulloa JJ (2008). Outcome analysis of mandibular condylar replacements in tumor and trauma reconstruction: a prospective analysis of 131 cases with long-term follow-up. J Oral Maxillofac Surg.

[41] Potter J, Dierks E (2008). Vascularized options for reconstruction of the mandibular condyle. Semin Plast Surg.

[42] Sanovich R, Mehta U, Abramowicz S, Widmer C, Dolwick MF (2014). Total alloplastic temporomandibular joint reconstruction using Biomet stock prostheses: the University of Florida experience. Int J Oral Maxillofac Surg.

[43] Mercuri LG, Edibam NR, Giobbie-Hurder A (2007). Fourteen-year follow-up of a patient-fitted total temporomandibular joint reconstruction system. J Oral Maxillofac Surg.

[44] Leandro LL, Ono HY, de Souza Loureiro CC, Marinho K, Guevara HG (2013). A ten-year experience and follow-up of three hundred patients fitted with the Biomet/Lorenz Microfixation TMJ replacement system. Int J Oral Maxillofac Surg.

